# Renal Paratyphoid

**Published:** 1918-07

**Authors:** 


**Renal Paratyphoid.** A. Gouget, De Prog. Med., Dec. 22, 1917, reports a man who, at the age of 19, had an acute nephritis, and at the age of 22, suffered a paratyphoid infection absolutely or, at least, predominantly, involving the kidneys. The only point in doubt was whether the albuminuria and the renal lesion itself had actually persisted from the time of the acute nephritis.

